# Heavy-Tailed Prediction Error: A Difficulty in Predicting Biomedical Signals of 1/*f* Noise Type

**DOI:** 10.1155/2012/291510

**Published:** 2012-12-05

**Authors:** Ming Li, Wei Zhao, Biao Chen

**Affiliations:** ^1^School of Information Science & Technology, East China Normal University, No. 500, Dong-Chuan Road, Shanghai 200241, China; ^2^Department of Computer and Information Science, University of Macau, Padre Tomas Pereira Avenue, Taipa, Macau

## Abstract

A fractal signal *x*(*t*) in biomedical engineering may be characterized by 1/*f* noise, that is, the power spectrum density (PSD) divergences at *f* = 0. According the Taqqu's law, 1/*f* noise has the properties of long-range dependence and heavy-tailed probability density function (PDF). The contribution of this paper is to exhibit that the prediction error of a biomedical signal of 1/*f* noise type is long-range dependent (LRD). Thus, it is heavy-tailed and of 1/*f* noise. Consequently, the variance of the prediction error is usually large or may not exist, making predicting biomedical signals of 1/*f* noise type difficult.

## 1. Introduction

Signals of 1/*f* noise type are widely observed in biomedical engineering, ranging from heart rate to DNA and protein, see, for example, [1–37], just to cite a few. Predicting such a type of signals is desired in the field [38–43]. A fundamental issue in this regard is whether a biomedical signal of 1/*f* noise type to be predicted is predicable or not.

The predictability of signals of non-1/*f* noise type is well studied [44–48]. However, the predictability of 1/*f* noise is rarely reported, to our best knowledge. Since many phenomena in biomedical engineering are characterized by 1/*f* noise [1–37], the predictability issue of 1/*f* noise is worth investigating.

Note that minimizing the mean square error (MSE) of prediction is a commonly used criterion in both theory and practical techniques of prediction, see, for example, [49–68]. Therefore, a sufficient condition for a biomedical signal *x*(*t*) to be predictable is that the variance of its predication error exists. If the variance of the predication error does not exist, on the contrary, it may be difficult to be predicted if not unpredictable. In the case of a signal being bandlimited, the variance of its predication error is generally finite. Consequently, it may be minimized and it is predictable. However, that is not always the case for biomedical signals of 1/*f* noise type.

Let *x*(*t*) be a biomedical signal in the class of 1/*f* noise. Then, its PDF is heavy-tailed, and it is LRD, see, for example, Adler et al. [69], Samorodnitsky and Taqqu [70], Mandelbrot [71], Li and Zhao [72]. Due to that, here and below, the terms, 1/*f* noise, LRD random function, and heavy-tailed random function are interchangeable.

Let *p*(*x*) be the PDF of a biomedical signal *x*(*t*) of 1/*f* noise type. Then, its variance is expressed by

(1) Var[x(t)]=∫−∞∞(x−μx)2p(x)dx,

where *μ*_*x*_ is the mean of *x*(*t*) if it exists. The term of heavy tail in statistics implies that Var[*x*(*t*)] is large. Theoretically speaking, in general, we cannot assume that Var[*x*(*t*)] always exists [72]. In some cases, such as the Pareto distribution, the Cauchy distribution, *α*-stable distributions [72], Var[*x*(*t*)] may be infinite. That Var[*x*(*t*)] does not exist is particularly true for signals in biomedical engineering and physiology, see Bassingthwaighte et al. [33] for the interpretation of this point of view.

Recall that a prediction error is a random function as we shall soon mention below. Therefore, whether the prediction error is of 1/*f* noise, or equivalently, heavy-tailed, turns to be a crucial issue we need studying. We aim at, in this research, exhibiting that prediction error of 1/*f* noise is heavy-tailed and accordingly is of 1/*f* noise. Thus, generally speaking, the variance of a prediction error of a biomedical signal *x*(*t*) of 1/*f* noise type may not exist or large. That is a reason why predicting biomedical signals of 1/*f* noise type is difficult.

The rest of this paper is organized as follows. Heavy-tailed prediction errors occurring in the prediction of biomedical signals of 1/*f* noise type are explained in Section 2. Discussions are in Section 3, which is followed by conclusions.

## 2. Prediction Errors of 1/*f* Noise Type

We use *x*(*n*) to represent a biomedical signal in the discrete case for *n* ∈ **N**, where **N** is the set of natural numbers. Let *x*_*N*_(*n*) be a given sample of *x*(*n*) for *n* = 0, 1,…, *N* − 1. Denote by *x*_*M*_(*m*) the predicted values of *x*(*n*) for *m* = *N*, *N* + 1, *N* + *M* − 1. Then, the prediction error denoted by *e*(*m*) is given by

(2) e(m)=∑m=NN+M−1x(m)−xM(m).

If one uses the given sample of *x*(*n*) for *n* = *N*, *N* + 1,…, 2*N* − 1 to obtain the predictions denoted by *x*_*M*_(*m*) for *m* = 2*N*, 2*N* + 1,2*N* + *M* − 1, the error is usually different from (2), which implies that the error *e*(*m*) is a random variable. Denote by *p*(*e*) the PDF of *e*(*m*). Then, its variance is expressed by

(3) Var[e(m)]=∑m=NN+M−1(e−μe)2p(e),

where *μ*_*e*_ is the mean of *e*(*m*).

Let *P* be the operator of a predictor. Then,

(4) xM(m)=PxN(n).

A natural requirement in terms of *P* is that Var[*e*(*m*)] should be minimized. Thus, the premise that Var[*e*(*m*)] can be minimized is that it exists.

It is obviously seen that Var[*e*(*m*)] may be large if *p*(*e*) is heavy tailed. In a certain cases, Var[*e*(*m*)] may not exist. To explain the latter, we assume that *e*(*m*) follows a type of heavy-tailed distribution called the Pareto distribution.

Denote by *p*_Pareto_(*e*) the PDF of the Pareto distribution. Then [73], it is in the form

(5) pPareto(e)=abaea+1,

where *e* ≥ *b*, *a* > 0, and *b* > 0. The mean and variance of *e*(*m*) are, respectively, expressed by

(6) μe=aba−1,Var(e)=ab2(a−1)2(a−2).

The above exhibits that Var[*e*(*m*)] does not exist if *a* = 1 or *a* = 2 and if *e*(*m*) follows the Pareto distribution.

Note that the situation that Var[*e*(*m*)] does not exist may not occur if *e*(*m*) is light-tailed. Therefore, the question in this regard is whether *e*(*m*) is heavy-tailed if a biomedical signal *x*(*n*) is of 1/*f* noise. The answer to that question is affirmative. We explain it below.

**Theorem 1**

Let *x*(*n*) be a biomedical signal of 1/*f* noise type to be predicted. Then, its prediction error is heavy-tailed. Consequently, it is of 1/*f* noise.

**Proof**

Let *r*_*xx*_(*k*) be the autocorrelation function (ACF) of *x*(*n*). Then,

(7) rxx(k)=E[x(n)x(n+k)],

where *k* is lag and *E* the mean operator. Let *r*_*MM*_(*k*) be the ACF of *x*_*M*_(*m*). Then,

(8) rMM(k)=E[xM(m)xM(m+k)].

Let *r*_*ee*_(*k*) be the ACF of *e*(*m*). Then,

(9) ree(k)=E[e(m)e(m+k)].

Note that

(10) ree(k)=E[e(m)e(m+k)]=E{[x(m)−xM(m)][x(m+k)−xM(m+k)]}=E[x(m)x(m+k)+xM(m)xM(m+k) −xM(m)x(m+k)−x(m)xM(m+k)]=rxx(k)+rMM(k)−rMx(k)−rxM(k).

In the above expression, *r*_*Mx*_(*k*) is the cross-correlation between *x*_*M*_(*m*) and *x*(*m*). On the other side, *r*_*xM*_(*k*) is the cross-correlation between *x*(*m*) and *x*_*M*_(*m*). Since *r*_*Mx*_(*k*) = *r*_*xM*_(*k*), we have

(11) ree(k)=rxx(k)+rMM(k)−2rxM(k).

Recall that *x*(*m*) is 1/*f* noise. Thus, it is heavy-tailed and hence LRD. Consequently, for a constant *c*_1_ > 0, we have

(12) rxx(k)~c1k−α (k→∞)  for  0<α<1.

On the other hand, the predicted series *x*_*M*_(*m*) is LRD. Thus, for a constant *c*_2_ > 0, the following holds:

(13) rMM(k)~c2k−β (k→∞)  for  0<β<1.

In (11), if *r*_*xM*_(*k*) is summable, that is, it decays faster than *r*_*x*_(*k*) or *r*_*M*_(*k*), it may be ignored for *k* → *∞*. In this case, *r*_*ee*_(*k*) is still non-summable. In fact, one has

(14) ree(k)~{c1k−α,0<α<β<1,c2k−β,0<β<α<1,(c1+c2)k−β,α=β. (k→∞),

On the other side, when *r*_*xM*_(*k*) is non-summable, *r*_*e*_(*k*) is non-summable too. In any case, we may write *r*_*ee*_(*k*) by

(15) ree(k)~ck−γ (k→∞)  for  0<γ<1.

Therefore, the prediction error *e*(*m*) is LRD. Its PDF *p*(*e*) is heavy-tailed according to the Taqqu's law. Following [72], therefore, *e*(*m*) is a 1/*f* noise. This completes the proof.

## 3. Discussions

The present result implies that cautions are needed for dealing with predication errors of biomedical signals of 1/*f* noise type. In fact, if specific biomedical signals are in the class of 1/*f* noise, the variances of their prediction errors may not exist or large [72]. Tucker and Garway-Heath used to state that their prediction errors with either prediction model they used are large [74]. The result in this paper may in a way provide their research with an explanation.

Due to the fact that a biomedical signal may be of 1/*f* noise, PDF estimation is suggested as a preparatory stage for prediction. As a matter of fact, if a PDF estimation of biomedical signal is light-tailed, its variance of prediction error exists. On the contrary, the variance of the prediction error may not exist. In the latter case, special techniques have to be considered [75–78]. For instance, weighting prediction error may be a technique necessarily to be taken into account, which is suggested in the domain of generalized functions over the Schwartz distributions [79].

## 4. Conclusions

We have explained that the prediction error *e*(*m*) in predicting biomedical signals of 1/*f* noise type is usually LRD. This implies that its PDF *p*(*e*) is heavy-tailed and 1/*f* noise. Consequently, Var[*e*(*m*)] may in general be large. In some cases [72], Var[*e*(*m*)] may not exist, making the prediction of biomedical signals of 1/*f* noise type difficult with the way of minimizing Var[*e*(*m*)].
